# Patient-centred outcome following endoscopic management of benign central airway obstruction

**DOI:** 10.1007/s00405-025-09230-1

**Published:** 2025-02-20

**Authors:** Mads Bøgh, Dalia Gustaityté Larsen, Matilde Lonka, Sten Schytte, Ulrik Pedersen, Søren Gade, Padraig O’Leary, Thomas Kjaergaard

**Affiliations:** 1 Otorhinolaryngology, Head and Neck Surgery, Aarhus University Hospital, Aarhus, Denmark; 2 Otorhinolaryngology, Head and Neck Surgery, Aalborg University Hospital, Aalborg, Denmark

**Keywords:** Therapeutic bronchoscopy, Central airway obstruction, Benign, Tracheobronchomalacia, Patient-relevant outcome, Postoperative complications

## Abstract

**Purpose:**

To examine patient-centred outcome following endoscopic treatment of central airway stenosis in terms of days alive and out of hospital (DAOH), need for re-intervention, and complications, with reference to aetiology of disease and applied treatment methodology.

**Methods:**

Analyses were based on data from consecutive adult patients treated endoscopically for benign central airway obstruction at Aarhus University Hospital from 2012 to 2022, with a minimum follow-up of one year. DAOH was calculated for 30 and 365 days. Complications were graded based on the Clavien-Dindo classification. Univariate and multivariate analyses were performed to identify predictors for DAOH, re-intervention and complications.

**Results:**

82 consecutive adult patients underwent endoscopic treatment during the period of inclusion, comprising a total of 175 dilatations, 42 benign tumour resections, and 67 stent insertions. Multiple interventions and short re-intervention intervals was more likely amongst patients reporting significant preoperative dyspnoea or requiring preoperative respiratory support, as well as patients treated with endoscopic insertion of silicone stents. The overall complication rate per procedure was 11.7%, and complications were more likely to occur in patients with high age, high BMI and comorbidity. Overall DAOH during the first year after intervention was 343 days, lowest amongst patients with tracheobronchomalacia or severe airway stenosis, and in those who underwent endoscopic stent insertion.

**Conclusion:**

Endoscopic treatment is a safe and viable intervention in the management of benign central airway obstruction in adults with few complications and a high overall outcome.

## Introduction

Benign central airway obstruction (bCAO) is a rare and complex condition, defined as any non-malignant stenosis of the trachea and/or main stem bronchi. Patients with bCAO may present with symptoms ranging from recurrent pneumonia and exertional dyspnoea to respiratory insufficiency and failure [1, 2].

In the majority of cases, bCAO is acquired and most often iatrogenic, i.e. post intubation or post tracheostomy tracheal stenosis. Other aetiologies include inflammatory diseases such as granulomatosis with polyangiitis, vascular & infectious diseases and benign neoplasms, as well as idiopathic subglottic stenosis [3, 4, 5, 6].

Most bCAO cases are suitable for endoscopic treatment, which generally has a high short-term success rate and low complication rate [7, 8, 9]. Nevertheless, endoscopic treatment often require re-intervention and is less effective in certain cases, such as idiopathic subglottic stenosis and cicatricial stenosis, i.e., cartilage deformity following tracheotomy [10, 11, 12]. In this context, bCAO can be considered as a chronic disease, imposing a long-term impact on patients quality of life. Consequently, the cumulative impact of repeated interventions, associated risks, and overall health outcomes must be considered when evaluating endoscopic treatment of bCAO. However, the literature is relative sparse in this field, and knowledge regarding the health impact of bCAO is limited.

The aim of this study was to examine the overall health impact of bCAO in patients treated with endoscopy. To address this issue, Days Alive and Out of Hospital (DAOH) was utilised. To our knowledge, this is the first time DAOH has been employed for patients with bCAO. We also assessed frequency and type of re-intervention as well as complications, based on the Clavien-Dindo classification.

## Methods

### Patient population and variables

Consecutive adult patients (≥ 18 years of age) receiving index endoscopic intervention for bCAO at Aarhus University Hospital (AUH), Denmark, during the period January 2012 to December 2022 were included. All patients were followed up through December 2023. The uptake area of AUH is approximately 1.5 million people, representing the Central Denmark Region. All endoscopic airway procedures in the region are centralized to AUH. Only individuals with no prior history of airway surgery, apart from tracheotomy, were eligible for inclusion. Electronic medical records were reviewed for demographic data, stenosis characteristics, symptoms, intervention urgency, type and frequency of interventions, and respiratory status prior to and following intervention (continuous positive airway pressure (CPAP)/non-invasive ventilation (NIV) and oxygen therapy, intubation), postoperative complications according to the Clavien-Dindo classification [13], severity of stenosis according to the Myer-Cotton classification [14], length of hospitalisation and overall survival. Comorbidity was assessed using the Charlson Comorbidity Index (CCI) [15]. Physical health was scored using the American Society of Anaesthesiologists Physical Status Classification (ASA) [16]. DAOH_365_ was calculated based on the first year following the index operation [17, 18]. Patient data were handled anonymously and in accordance with regional research permit (1-45-70-16-23).

### Surgical procedures

Endoscopic procedures included balloon dilation with mucosal incision, tumour resection, and stent insertion. Balloon dilation was mainly performed by sequential dilations with balloons of increasing size, predominantly combined with mucosal incision and steroid injection. Endoscopic resections were conducted using cold steel, CO2 laser, or electrocautery. Applied stents were silicone stents by Novatech (La Ciotat, France), either I or Y configuration, and always tailored based on type and location of the obstruction as well as patient-specific factors. Stents were always placed endoscopically by rigid bronchoscopy according to previously described techniques [19].

Postoperative antibiotics and systemic steroids were administered routinely, in all patients. All patients were hospitalized following intervention and discharged when fit, according to routines, usually the first postoperative day.

### Outcomes

Our primary outcome was patient-centred outcome following intervention, based on DAOH_365_, a metric representing the number of days a patient is alive and not hospitalised within a specified period. It reflects the effectiveness of a treatments or interventions by emphasising the quality of life by highlighting the time patients spend outside the hospital instead of just survival, thereby indicating recovery and overall well-being of the patient.

Secondary outcomes were need of re-intervention following index-surgery and time to re-intervention, short-term complications graded by the Clavien-Dindo classification [13]. This is a standardised system for grading postoperative complications based on the required therapeutic intervention. The grades are as follows: I (no pharmacological or surgical intervention), II (requiring pharmacological treatment or blood transfusion), III (requiring surgical, endoscopic, or radiological intervention, IV (life-threatening complications requiring ICU management), and V (death).

### Statistical analysis

Differences in frequency of re-intervention between subgroups was assessed by Kaplan-Meier curves and the log-rank test. Unadjusted mean with 95% confidence interval (CI) was calculated for DAOH_365_. Effectiveness of an intervention, represented by the time intervals between interventions, was compared between subgroups by the Welch two-sample t-test. Predictors for postoperative complications, multiple interventions, and DAOH were identified based on univariate and multivariate analyses. Statistical significance was defined as *p* < 0.05.

## Results

**Table 1:** Clinical characteristics (*n = 82*)

Variable^a^	Number of patients	
**Age (years)**	54 (17)	
**BMI**	27.7 (7.1)	
**Male Sex**	33 (40.2%)	
**Aetiology**		
Traumatic^b^	27 (32.9%)	
Idiopathic	26 (31.7%)	
Inflammatory	16 (19.5%)	
Benign Neoplasm/extern compression	13 (15.9%)	
**Alcohol Consumption**		
More than 10 drinks per week	7 (8.5%)	
**Smoking**		
Never Smoked	47 (57.3%)	
Ex-smoker	25 (30.5%)	
Smoker	10 (12.2%)	
**Respiratory Status**		
Respiratory Support	11 (13.4%)	
Dyspnoea	69 (84.2%)	
Chronic Coughing	13 (15.9%)	
Recurrent pneumonias	12 (14.6%)	
**Affected Airway Segments**		
Trachea	71 (86.6%)	
Bronchi	18 (22.0%)	
Carina	5 (6.1%)	
≥ 2 Affected Segments	9 (11.0%)	

^a^Continuous data are presented as means (SD) and categorical data as numbers

^b^Primarily iatrogenic (96.3%)

### Patient characteristics

In total, 82 patients were included in the study with a median follow up was 663 days (range: 3–3946 days). Overall, 67 stent insertions, 175 balloon dilations and 42 endoscopic tumour resections were performed during the period. Clinical characteristics are shown in Table 1. The majority of traumatic bCAO were related to tracheotomy (17), and intubation (5), inflammatory conditions were mainly represented by granulomatosis with polyangiitis (13), whereas benign neoplasms represented a mix of lesions.

At the time of the index intervention, 11 patients were receiving respiratory support: oxygen therapy (1), CPAP/NIV (4), intubation (6). Stridor prior to the index intervention was observed in 35 patients (42.7%).

Tracheobronchomalacia was found in 12 patients, either in combination with a fixed stenosis (5) or as a solitary pathology (7).

The index operation was classified as urgent (performed within 24 h) in seven cases and semi-urgent (performed within seven days) in one case.

**Fig. 1:**
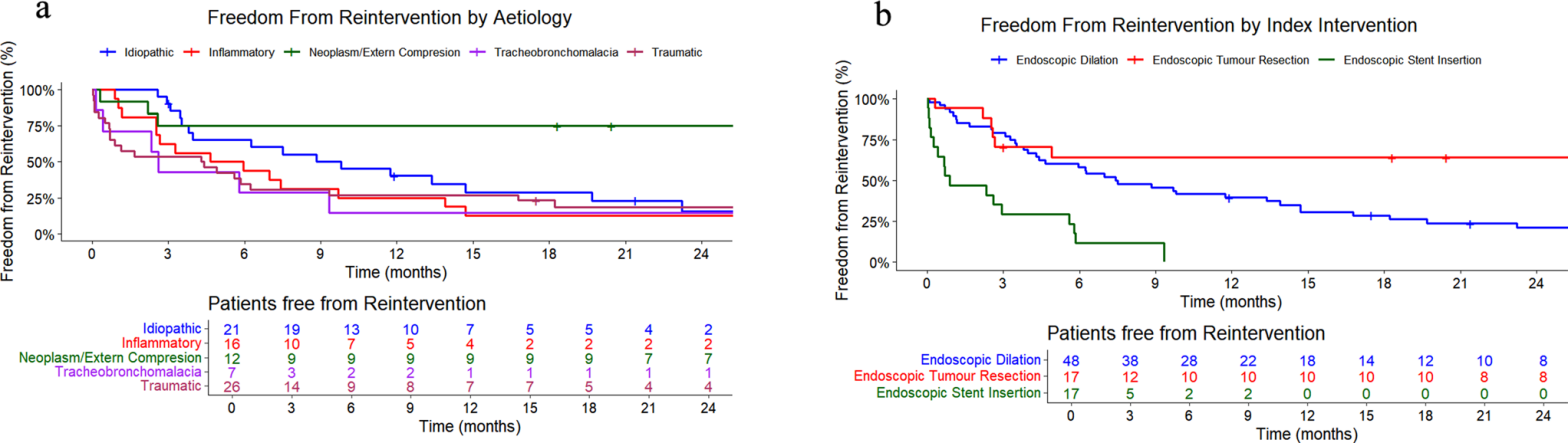
Freedom from re-intervention for patients stratified by (**a**) aetiology and (**b**) index intervention

Prior to the index intervention, four patients were classified as ASA I (4.9%), 42 as ASA II (51.2%), 43 as ASA III (52.4%) and three as ASA IV (3.7%). One was unknown.

### Days alive and out of hospital

Mean DAOH365 was 343 days. When stratified by index intervention, mean DAOH_365_ was significantly lower in patients treated with endoscopic stent insertion (288 days) compared to patients treated with dilation (355 days, *p* = 0.002), and endoscopic tumour resection (362 days, *p* = 0.03).

Lower DAOH365 was observed in patients who required urgent intervention (DAOH_365_ = 209, *p* < 0.001), had excessive alcohol consumption (DAOH_365_ = 252, *p* < 0.001, or needed preoperative respiratory support (DAOH_365_ = 246, *p* < 0.001). Furthermore, DAOH_365_ was found to be significantly lower in patients in whom the stenosis involved the bronchi (DAOH_365_ = 300, *p* = 0.004), the tracheal carina (DAOH_365_ = 224, *p* < 0.001), or multiple airway segments i.e. trachea and one or both main stem bronchi *(*DAOH_365_ = 245, *p* < 0.001). Solitary tracheobronchomalacia was also associated with a significantly lower DAOH_365_ of 211 days, *p* = 0.01. No difference in DAOH_365_ was observed between patients with Myer-Cotton grade I stenosis (0–50%) compared to patients with grade II-IV (51–100%), *p* = 0.1.

### Re-interventions

During the first year after the index intervention 65,9% of the patients required re-intervention. After two years, 81,7% had undergone re-intervention. Mean time to re-intervention after index surgery was 474 days. As shown in Fig. 1b, airway stenting was associated with significantly shorter re-intervention intervals (137.3 days) compared to ballon dilation (365.9 days), *p* < 0.001, and tumour resection (652.1 days), *p* < 0.001. A significant difference was observed between balloon dilation and endoscopic tumour resection, *p* = 0.01. No difference was observed in time to re-intervention following index intervention in patients with Myer-Cotton grade I stenosis compared to the those with grade II-IV, *p* = 0.6.

Mean re-intervention interval was 534 days for all patients. This interval was 331 days in patients with traumatic bCAO, *p* = 0.4, and 536 days in patients with inflammatory bCAO, *p* = 0.4. The re-intervention interval was significantly shorter in patients with idiopathic bCAO, 367 days, *p* < 0.001, while it was significantly longer in patients with intraluminal tumours and/or external compression, namely 1450 days, *p* = 0.01. Patients with solitary tracheobronchomalacia also experienced significant shorter re-intervention intervals of 207 days, *p* = 0.01.

The mean number of interventions per patient during the first year was 1.9. Beyond the first year, the mean number of interventions per patient per year decreased significantly to 0.5 (*p* < 0.001). On average, patients underwent 1.7 interventions for the remainder of the observation period following the initial year.

As shown in Table 2, odds for multiple interventions per year were found to be significantly higher in patients who received respiratory support prior to the first intervention or reported dyspnoea at rest.

**Table 2:** OR for potential risk factors for two or more interventions per year (*n* = 82)

	OR (95% CI)	*p*-value
**Patient Characteristics and Condition**		
Female Sex	1.4 (0.5–3.8)	0.5
Age < 65 years	1.0 (0.1–2.9)	1.0
Current or Previous Smoker	1.3 (0.5–3.5)	0.6
Excessive Alcohol Intake	3.9 (0.8–19.2)	0.1
BMI under 25	1.1 (0.4–2.9)	0.9
Respiratory Support	**6.0 (1.6–23.2)**	**0.01**
Dyspnoea at rest	**3.6 (1.2–10.8)**	**0.03**
CCI Score ≥ 3	1.0 (0.4–2.6)	1.0
ASA Score ≥ 3	1.7 (0.7–4.5)	0.3
**Lesion and Procedure Characteristics**		
Lesion Involving Bronchi	1.9 (0.6–5.8)	0.3

In 15.9% of the cases, endoscopic intervention was insufficient to reach a satisfactory clinical result, and tracheal/cricotracheal resection (*n* = 7) or a tracheotomy (*n* = 6) was performed. The highest failure rates for endoscopic therapy were seen in patients with traumatic bCAO, *p* < 0.001.

### Complications

As shown in Table 3, insertion of airway stents was associated with a higher complication rate (28.4%) compared to tumour resections (11.9%) and balloon dilations (5.1%), *p* < 0.001 and *p* = 0.04, respectively. No significant difference was observed between dilation and endoscopic resection, *p* = 0.1. Overall rate of complications per intervention was 11.7%.

Complication rates were significantly higher for traumatic bCAO (20,9%) and solitary tracheobronchomalacia (28,6%), compared to inflammatory bCAO (8.0%), idiopathic bCAO (3.9%) and benign tumours (3.7%), *p* = 0.02.

An increased risk of perioperative complications was seen in patients aged 65 or older (OR = 4.0, *p* = 0.009), with a BMI of more than 25 (OR = 3.0, *p* = 0.04), needing preoperative respiratory support (OR = 6.5, *p* = 0.008) and with a CCI score above 2 **(**OR = 5.3, *p* = 0.002). No significant difference in risk of complications was registered between patients with My-Cotton grade II-IV and grade I (OR = 1.2, *p* = 0.8).

Based on the Clavien-Dindo classification for grading complications, one patient experienced grade I complications (requiring out-patient follow-up), 13 patients experienced grade II complications (requiring hospitalisation), 16 patients experienced grade III complications (requiring re-intervention), and 3 patients experienced grade IV complications (requiring ICU treatment and respiratory support).

**Table 3:** Complications associated with endoscopic interventions (*n = 82*)

	Stent		Resection		Dilation	
	*n*	%	*n*	%	*n*	%
**Grade of Complication** ^**a**^						
I	1	1.5%	0	-	0	-
II	5	7.5%	2	4.8%	5	2.9%
III	10	14.9%	3	7.1%	3	1.7%
IV	2	3.0%	0	-	1	0.6%
V	1	1.5%	0	-	0	-
**Type of Complication**						
Pneumonia	3	4.5%	0	-	3	1.7%
Stent Migration	8	11.9%	0	-	0	-
Stent Occlusion	4	6.0%	0	-	0	-
Bleeding	0	-	1	2.4%	0	
Respiratory Insufficiency	4	6.0%	2	4.8%	2	1.1%
Restenosis	0	-	2	4.8%	4	2.3%

The only fatal outcome was registered in a patient treated with endoscopic stent insertion, who experienced respiratory insufficiency shortly after the procedure. Thus, the mortality rate of stent insertions was 1.5%.

## Discussion

The preference of intervention for bCAO is influenced by various factors, mainly aetiology, risk profile and anticipated outcomes. Consequently, the most appropriate intervention may vary between two patients with similar aetiologies. Thus, insights into treatment efficacy and related risks is of paramount importance in decision making.

To our knowledge, this is the first study to report on DAOH in patients treated endoscopically for bCAO. DAOH is a patient-centred outcome measure reflecting hospitalisations and mortality following an intervention, in particular surgery. It provides a comprehensive view of the effectiveness of a given treatment, capturing survival and quality of life outside the hospital [17, 18]. With reference to bCAO, which, in many cases represent a chronic, recurrent condition, it seems relevant to impose the DAOH measure. In our cohort, we found a mean DAOH_365_ of 343 meaning that the average patient was either hospitalised or dead for 22 days within the first year following the index intervention. Not surprisingly, DAOH varied amongst subgroups with the lowest outcome in the stent-group (DAOH_365_ 288) and the highest outcome in the dilatation and tumour resection subgroups (DAOH_365_ 352 and 362 respectively). Likewise, disease severity, reflected by urgency of the procedure and preoperative respiratory support, also affected DAOH in a negative way. For comparison, studies have reported DAOH_365_ of 273 in patients undergoing left ventricular assist device implantation for chronic heart failure [20] and 355 days in patients undergoing liver transplant [21]. However, most studies employing DAOH on a cohort of patients with non-malignant disease(s) include only data for the first 90 or 180 days, hindering direct comparison of the long-term outcome [22, 23].

Although the likelihood for re-intervention after endoscopic treatment for bCAO is relatively high, re-intervention intervals were generally long (mean interval 474 days). Not surprisingly, re-intervention intervals varied significantly by aetiology and intervention type, with airway stenting having the shortest intervals.

Although shorter intervals in the stent population may be partly explained by local recommendations i.e. shift of stent every four to six months, our findings emphasise that treatment with silicone stents indeed poses a higher treatment load than other endoscopic interventions. This aligns with previous studies, namely Karush et al. [8], examining 243 procedures of silicone stent insertion in 63 patients, presenting a median re-intervention interval of 104 (IQR 167) days, and Jeong et al. [24] and Dalar et al. [25], presenting a mean re-intervention interval of 79.5 days and 162 days respectively.

Taken together, we present somewhat higher overall re-intervention intervals compared to previous publications, with ranges between 195 and 343 days [26, 27].However, direct comparison is not meaningful, due to the inherent variability, illustrated in our data as well as by others [28].

Overall, a single intervention was sufficient to achieve disease control in 19.5% of cases, with the highest percentage in the tumour resection subgroup (47.1%). This is in line with other studies, which report the use of a single modality in up to 40% of cases [29, 30]. However, as noted earlier, direct comparison is limited.

The “failure rate” of endoscopic intervention, reflected by the proportion of conversions from endoscopic approach to open surgery (i.e., tracheal and cricotracheal resection) or tracheotomy, was low. Ultimately, 15.9% of patients were treated by an open approach. Not surprisingly this was most evident in patients with traumatic/iatrogenic bCAO which is in line with findings from others such as Özdemir et al. [31], presenting a surgical failure rate of 17.4%.

Although re-intervention following endoscopic surgery is indeed very likely, as opposed to open surgery [7, 30, 32, 33, 34, 35, 36] the overall procedure-related complications were low. We found an overall complication rate of 11.7% per intervention, highest in the airway stent subgroup (28.4%). This is relatively low compared with published results (range 30-84.5%) [30, 31, 37]. Based on the Clavien-Dindo Classification, the majority of complications (88.2%) were relatively mild and only 8.9% requiring respiratory support or ICU-treatment. The overall mortality rate was low, 0.4%. As expected, treatment with silicone stent was associated with a higher risk of postoperative complications compared to non-stent procedures.

Apart from the inherent limitations of a retrospective design, our study is somewhat limited by the relatively small sample size, although large in the context of previous publications. Further, the heterogeneity of our cohort limits direct comparability with other studies.

## Conclusion

To summarise, endoscopic intervention, specifically dilation and tumour resection, appears to be a safe and effective intervention for adult patients with bCAO, imposing a limited impact on overall health, presented by DAOH. Amongst endoscopic procedures, insertion of endoluminal silicone stents is associated with the greatest impact on overall health and should thus be reserved for complex cases where other endoscopic interventions have proven insufficient and open surgery is not a viable option.

## Abbreviations

ASA: American Society of Anaesthesiologists Physical Status Classification System
AUH: Aarhus University Hospital
bCAO: Benign Central Airway Obstruction
CCI: Charlson Comorbidity Index
CI: Confidence Interval
CPAP: Continuous Positive Airway Pressure
DAOH: Days Alive and Out of Hospital
NIV: Non-Invasive Ventilation
